# *In vivo* pharmacological activity and biodistribution of S-nitrosophytochelatins after intravenous and intranasal administration in mice

**DOI:** 10.1016/j.niox.2016.06.006

**Published:** 2016-09-30

**Authors:** Lamia Heikal, Anna Starr, Gary P. Martin, Manasi Nandi, Lea Ann Dailey

**Affiliations:** Institute of Pharmaceutical Sciences, Faculty of Life Science & Medicine, King’s College London, 150 Stamford Street, London, SE1 9NH, UK

**Keywords:** Nitric oxide, S-nitrosoglutathione, S-nitrosophytochelatins, Phytochelatins, Biodistribution, S-nitrosothiols

## Abstract

S-nitrosophytochelatins (SNOPCs) are novel analogues of S-nitrosoglutathione (GSNO) with the advantage of carrying varying ratios of S-nitrosothiol (SNO) moieties per molecule. Our aim was to investigate the *in vivo* pharmacological potency and biodistribution of these new GSNO analogues after intravenous (i.v.) and intranasal (i.n.) administration in mice. SNOPCs with either two or six SNO groups and GSNO were synthesized and characterized for purity. Compounds were administered i.v. or i.n. at 1 μmol NO/kg body weight to CD-1 mice. Blood pressure was measured and biodistribution studies of total nitrate and nitrite species (NOx) and phytochelatins were performed after i.v. administration. At equivalent doses of NO, it was observed that SNOPC-6 generated a rapid and significantly greater reduction in blood pressure (∼60% reduction compared to saline) whereas GSNO and SNOPC-2 only achieved a 30–35% decrease. The reduction in blood pressure was transient and recovered to baseline levels within ∼2 min for all compounds. NOx species were transiently elevated (over 5 min) in the plasma, lung, heart and liver. Interestingly, a size-dependent phytochelatin accumulation was observed in several tissues including the heart, lungs, kidney, brain and liver. Biodistribution profiles of NOx were also obtained after i.n. administration, showing significant lung retention of NOx over 15 min with minor systemic increases observed from 5 to 15 min. In summary, this study has revealed interesting *in vivo* pharmacological properties of SNOPCs, with regard to their dramatic hypotensive effects and differing biodistribution patterns following two different routes of administration.

## Highlights

• First *in vivo* characterization of S-nitrosophytochelatins cardiovascular effects.
• Increase in SNOPC-peptide lengths correlate with enhanced release kinetics.
• SNOPC peptide length correlates with extent of reduction in blood pressure.
• Evidence of S-nitrosothiol accumulation in RBCs after i.v. administration.
• First evidence of biodistribution kinetics of SNOPC metabolites *in vivo.*

## Graphical abstract

**Figure:**
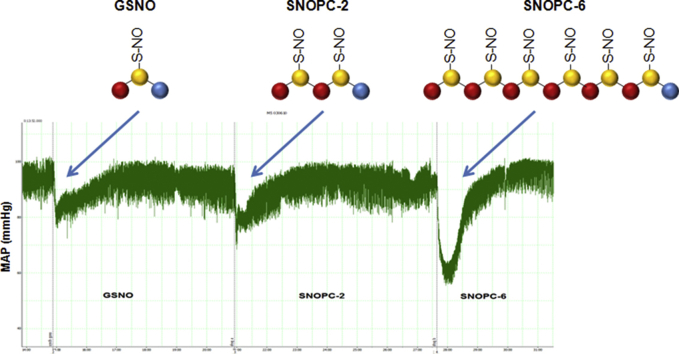

## 1 Introduction

Nitric oxide (NO) is an extremely versatile signalling molecule with an extraordinarily diverse array of physiological functions. Disruption to endogenous NO synthesis pathways is a common underlying factor in a variety of pathological conditions, importantly endothelial dysfunction [1], [2]. Reduced vascular levels of NO production are responsible for a variety of cardiovascular disorders contributing towards elevated blood pressure, vascular remodelling, and thrombotic events [2]. Exogenous administration of NO *via* NO-donor molecules has been explored as an attractive therapeutic strategy to treat not only cardiovascular disorders [3], [4], underpinned by endothelial dysfunction, but also a variety of other pathological conditions, including cancer, infection, osteoporosis, and wound healing [5], [6], [7], [8].

**Fig. 1:**
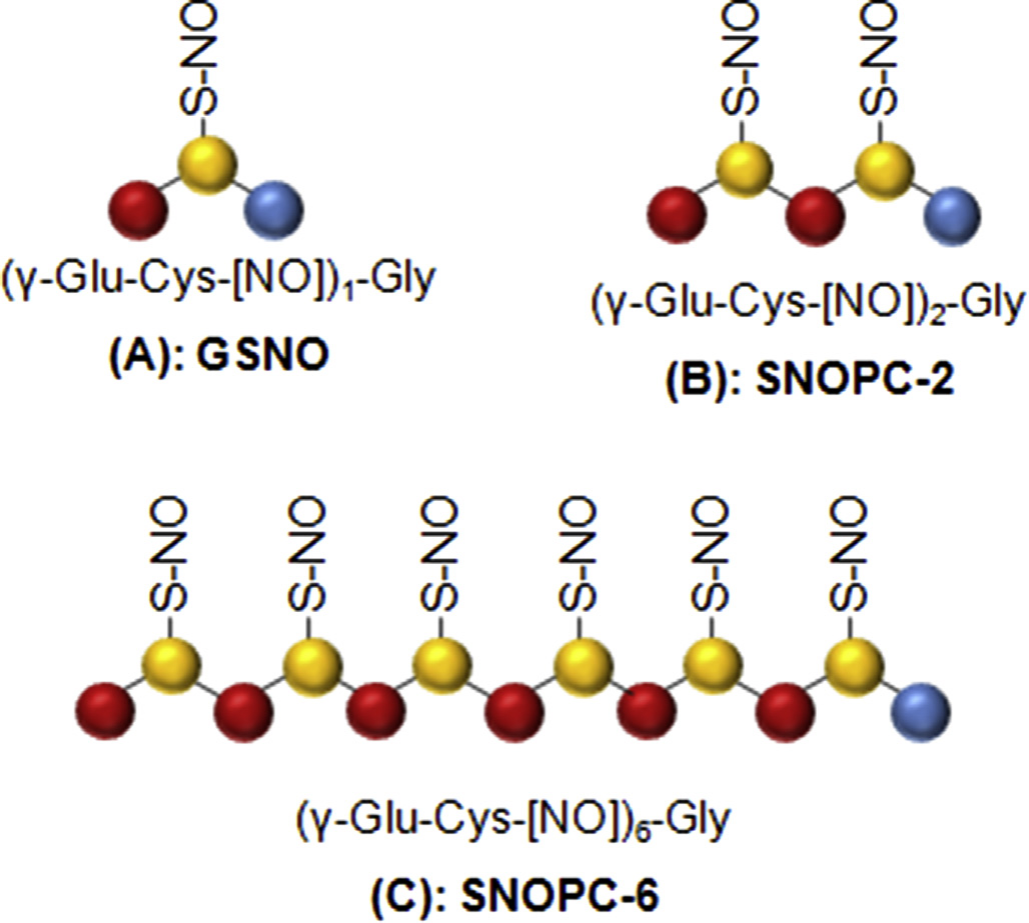
Schematic structures of (A) GSNO, (B) SNOPC-2 and (C) SNOPC-6.

The key to successful therapeutic use of NO-donors is achieving targeted NO release and a therapeutically suitable pharmacokinetic profile [9]. Due to the nature of NO as a small, extremely labile and reactive molecule, this objective has proven very challenging to achieve. Interest in the development of appropriate NO delivery systems has increased rapidly across a diverse field of applications [9]. S-nitrosothiols represent a class of NO donors where most have been previously synthesized as S-mono-nitrosothiols based on two different thiol moieties, either penicillamine or cysteine. However, the current trend is the development of di- or poly-S-nitrosothiols in order to increase the payload of compounds releasing NO, thus limiting the drug concentration [10], [11]. Poly S-nitrosothiols that have been synthesized include *S*,*S*′-dinitrosobucillamine (BUC(NO)_2_), which combines in its structure two *S*-mono-nitrosothiols, *S*-nitroso-*N*-acetylpenicillamine and *S*-nitroso-*N*-acetylcysteine [10]. *S*-nitroso-β-cyclodextrins; a compound that combines photochemically and thermally induced NO release with drug carrier ability have been also synthesized where six 6-mono- and 6-multi-*S*-nitroso-β-cyclodextrins (SNO-βCDs) were characterized in terms of their SNO content [12]. Poly-S-nitroso albumin has been developed as a safe and potent multifunctional antitumor agent [13].This study focuses on the *in vivo* activity and pharmacokinetic profiles of a new class of oligopeptide-based NO delivery systems known as S-nitroso phytochelatins (SNOPCs; Fig. 1).

Phytochelatins (PCs) are cysteine-rich oligopeptides produced in plants in response to heavy metals, especially Cd^2+^, contamination found in soil [14]. Their physiological function is to sequester reactive heavy metals *via* chelation with their cysteine thiol groups, thereby playing a protective role in detoxification. In this way, they are analogous to metallothioneins in mammals. Structurally, PCs are similar to glutathione (GSH), with a primary sequence of (γ-Glu-Cys) _n_ –Gly where usually n = 2–5, but may reach up to 11 in higher varieties of plant species and microorganisms [14], [15]. Recent studies have also shown that endogenous NO can react with PCs in plant tissues to produce endogenous mono S-nitrosylated PCs [16]. Interestingly, *in vivo* mono-S-nitrosylation of PCs occurs in a site specific manner, selectively on the single cysteine thiol nearest the N-terminal group. It is thought that this specific S-nitrosylation pattern plays an important role in cell signalling, but to date, little information on the exact nature of such pathways exists [16], [17], [18].

SNOPCs may also be utilized as oligopeptide-based NO delivery systems [8], [19]. Under *in vitro* conditions, full S-nitrosylation may be achieved, creating an NO delivery system that carries multiple moieties of S-nitrosothiol groups (SNO). Using isolated rat aortic rings, we have previously shown that SNOPCs carrying two-, four or six moieties of SNO (SNOPC-2, -4 or -6) are able to elicit a strong vasodilatory response, equivalent to GSNO at equal molar concentrations of SNO, and more potent than GSNO at equal molar concentrations of compound. However, we observed that SNOPCs are prone to a more rapid physicochemical degradation compared to GSNO and this reduced their biological activity in protracted *in vitro* experiments [19].

As SNOPCs were observed to be excellent transnitrosating agents under *in vitro* conditions [19], [20], we were interested in examining their *in vivo* pharmacological activity and biodistribution profiles after intravenous (i.v.) injection. Further, due to a potential therapeutic benefit of inhaled NO donor compounds in diseases such as pulmonary hypertension [21], [22], the pharmacokinetics and biodistribution of SNOPCs and GSNO was investigated after intranasal (i.n.) administration. Thus, the aim of this study was to evaluate the pharmacological activity of the SNOPCs as NO delivery systems after i.v. administration and characterize the pharmacokinetic profile of these compounds (both the NO component as well as the oligopeptides carriers) after i.v. and i.n. administration.

## 2 Materials and methods

### 2.1 Materials

Phytochelatins PC-2 (Mol. wt. = 540.6 Da) and PC-6 (Mol. wt. = 1468.6 Da) were obtained from ANASPEC (Mountain View, CA, USA). Fluorescent-labelled glutathione, PC-2 and PC-6 (conjugated with 7-methoxy coumarin) were obtained from PEPCEUTICALS Limited (Leicestershire, UK). Coomassie Plus (Bradford) Protein Assay reagent was purchased from Thermo Fisher Scientific (Rockford, USA). All other analytical grade chemicals and reagents were purchased from Sigma–Aldrich (Dorset, UK). Mice (male CD-1, 8 weeks old) were purchased from Charles River UK Ltd.

### 2.2 Synthesis and characterization of GSNO and SNOPCs

Stock solutions of GSNO, SNOPC-2 and -6 were prepared as previously described [20]; an equal volume of acidified nitrite solution (NaNO_2_/0.9 M HCl) was added to the appropriate glutathione or phytochelatin solution (in 0.9% saline) using equimolar ratios of thiol groups:NaNO_2_ under conditions where light was excluded. The final pH of the solutions was maintained at 7.4 using 4 mol/L NaOH in 0.1 mol/L Tris buffer. S-nitrosation efficiency (%) was quantified and calculated using UV/Vis absorbance at λ = 334 nm (Cary Varian-300; UK). S-nitrosation efficiencies were 100, 95 and 89% for GSNO, SNOPC-2 and SNOPC-6, respectively. *GSNO and *SNOPCs (*peptides labelled with 7-methoxycoumarin) were S-nitrosated according to the same method and it was determined that the label did not interfere with S-nitrosation efficiency (*GSNO: 98%, *SNOPC-2: 94%, and *SNOPC-6: 88%). All solutions were stored under light exclusion on ice and were administered within 30 min of preparation.

### 2.3 In vivo studies

All animal experiments were performed under UK Home Office approval according to the Animals Scientific Procedures Act, 1986. Studies were designed and conducted in accord with the ARRIVE guidelines. Experiments were conducted at approximately the same time of day to account for diurnal variation. Drug administration was randomized and unblinded, but data was analyzed by a blinded investigator. Commercially available CD-1 adult male mice (∼0.025 Kg body weight) were used for all studies and acclimatized in home cages for 1 week prior to experimentation with *ad libitum* access to food and water and on a 12 h light-dark cycle [23]. Routes of drug administration, surgical protocols and data analysis are detailed below. A total of 180 mice were used, complying with the permitted animal usage outlined in the corresponding Home Office licence. The severity of all procedures was classified as mild.

#### 2.3.1 Haemodynamic measurement of the hypotensive effect of GSNO and SNOPCs in whole animal models

*In vivo* blood pressure was monitored in anaesthetized animals as previously described [24]. A total of 12 wild type male mice were used (n = 3/group) where the groups represented the vehicle (0.9% saline) control, GSNO, SNOPC-2 and SNOPC-6 treatments, respectively. Mean arterial pressure was recorded in anaesthetized mice (2% isoflurane in air) on a homeothermically controlled heating blanket (Harvard Instruments, Edenbridge, UK). A 1.2F microtip pressure catheter (Scisense, Transonic Systems, Ithaca, NY, USA) was inserted into the right carotid artery and basal arterial pressure was recorded for 5 min. Test drug solutions (50 μL containing 500 μmol/L NO ≈ 1 μmol NO/kg body weight) or control (saline) solution were directly injected into the jugular vein using a 30 gauge needle inserted through the underlying muscle. Arterial blood pressure was recorded continuously for a further 5 min following drug administration using a PowerLab data acquisition system and Chart 7 software (ADInstuments, Oxford, UK).

#### 2.3.2 Biodistribution studies after i.v. administration

A total of 72 mice were used (n = 3/time point/group) where the groups represented the vehicle (0.9%saline) control, GSNO, SNOPC-2 and SNOPC-6 treatments, respectively. A time course study (0, 1, 3, 5, 10 and 15 min) was carried out to characterize the biodistribution of NO metabolites (total NOx and nitrite) or labelled peptides in different organs after i.v. injection. I.V. injection of a single volume (50 μL) of test solution in saline (containing 500 μmol/L NO ≈ 1 μmol NO/kg body weight) or control (saline) solution was given into the lateral tail vein of the mice while under anaesthesia (2% isofluorane). At the appropriate time point, animals were exsanguinated by terminal bleed under anaesthesia (2% isofluorane). Blood samples were collected in heparinized tubes (100 U/mL) and centrifuged at 1200 g for 5 min at 4 °C to separate the plasma from the cellular fraction. Without perfusing the organs, the heart, liver and lung were dissected within 5 min and snap frozen in liquid nitrogen and stored at −80 °C until required for further processing.

#### 2.3.3 Biodistribution studies after i.n. administration

A total of 48 mice were used (n = 3/group/time point) where the groups represented vehicle (0.9%saline) control, GSNO, SNOPC-2 and SNOPC-6 treatments (n = 3 per group), respectively. A time course study (0, 1, 5 and 15 min) was carried out to characterize the NOx biodistribution after i.n. administration of compounds. Prior to drug administration, mice were anaesthetized by inhaling 2% isoflurane in air *via* a nose cone. A single volume (20 μL) of test solution in saline (containing 1.25 mmol/L NO ≈ 1 μmol NO/kg body weight) or control (saline) solution was then administered drop wise into the nasal cavity using a micropipette after removing the mouse momentarily from the anaesthetic mask cone and holding it in an upright position. At the appropriate time point animals were exsanguinated by terminal bleed under anaesthesia. Blood and organ samples were collected and processed as described in section 2.4.

#### 2.3.4 Quantification of fluorescently labelled PC oligopeptides

The fate of the PC oligopeptide backbone was assessed by direct quantification of 7-methoxycoumarin present in 100 μL plasma or tissue homogenate collected at specified time points (0, 1, 5 and 15 min) post injecting animals *in vivo* with 7-methoxycoumarin labelled drugs (n = 3/group/time point, total of 48 mice). The fluorescence in plasma or tissue homogenate was measured at λ_ex_ = 340 nm and λ_em_ = 400 nm. Following subtraction of background fluorescence from control tissues, the amount of fluorescent-labelled oligopeptide was calculated from a linear calibration curve constructed from known concentration of 7-methoxycoumarin-labelled compounds (linear range: 0.1–1.5 μmol *GSNO, *SNOPC-2 and *SNOPC-6, respectively). Values were normalized for total amount of protein (mg) and expressed as percentage of the initial dose (% ID/mg protein). Note: Because compounds were dosed at equimolar NO concentrations, administered doses of the labelled PCs were: *GSNO: 1.00 μmol/kg, *SNOPC-2: 0.50 μmol/kg, *SNOPC-6: 0.17 μmol/kg.

### 2.4 Quantification of total NOx and S-nitrosothiols

Prior to quantification, tissue samples were thawed, weighed and homogenized at 100 mg tissue/mL in phosphate buffered saline (PBS; pH 7.4) using a Silverson model L4RT laboratory homogenizer. Homogenates were centrifuged at 13,000 g for 20 min at 4 °C to remove tissue debris. Whole blood samples were fractionated into plasma (serum + platelets) and blood cells (red and white blood cells) by centrifugation at 1200 g. Cellular blood components were lysed with lysis buffer (5 mmol/L Na o-vanadate, 10 mg/mL benzamidine, 1 mmol/L phenylmethylsulfonyl fluoride (PMSF), 10 mmol/L Tris HCl, 5 mmol/L ethylenediamine tetraacetic acid (EDTA), 50 mmol/L NaCl pH 7.4 and 3.5 mmol/L SDS) at a volume ratio of 1:5 cells: lysis buffer. These samples were centrifuged at 13,000 g for 20 min at 4 °C. Protein content of each sample was quantified using the Bradford assay [25].

Quantification of total NOx was performed according to a method described by Verdon et al. [26]. Briefly, samples were de-proteinated by mixing the sample with 0.3 mol/L NaOH and aqueous 0.27 mol/L ZnSO_4_ at a volume ratio of 1:1:2. NOx was determined in 50 μL de-proteinated sample in the form of nitrite using the colorimetric Griess method [27], [28]. The amount of nitrite in each sample was calculated from a linear calibration curve of known nitrite concentrations (linear range: 1–100 μmol/L) and normalized for total amount of protein (mg), quantified using the Bradford assay. The final concentration, expressed as μM nitrite/mg protein, represents the total NOx in the system. Any remaining nitrite in the infusate (drug solution administered) was not measured as we were more interested in *in vivo* changes in NOx levels in untreated versus different treated groups at different time points measured but we have previously characterized this [20].

Total S-nitrosothiol compounds (RSNO) were quantified in plasma and the blood cell fraction only by gas-phase chemiluminescence reaction with ozone [29], [30]. Briefly, blood samples were treated with 5 mmol/L N-ethylmaleimide (NEM) shortly after withdrawal from mice to chelate copper and prevent RSNO degradation. 300 μL of the test sample was quickly injected into a purge vessel containing 2 mL potassium iodide solution (50 mg/mL) and 6 mL glacial acetic acid under an NO-free N2 atmosphere. The tri-iodide solution formed reduced RSNOs to NO gas which was detected by using Eco Physics NO analyzer (model CLD88). Plasma and blood cell RSNOs were quantified by the NO signal peak area of the samples. Data were quantified using NOanalyser software.

### 2.5 Data analysis

As each time point required a terminal bleed for blood and tissue harvest, n = 3 was the minimum number of animals to be used to report that PK profile over time whilst still complying with the appropriate animal usage as outlined within the UK Home Office animal licence used for this body of work. All data were analyzed using Graph Pad Prism 4 software. One-way or two-way analysis of variance (ANOVA) followed by Bonferroni’s multiple comparison post-test was used. The following system was used to denote the p-value: *p < 0.05, **p < 0.01, ***p < 0.001.

## 3 Results and discussion

### 3.1 SNOPC-6 is more potent than SNOPC-2 and GSNO at equivalent administered doses of NO

**Fig. 2:**
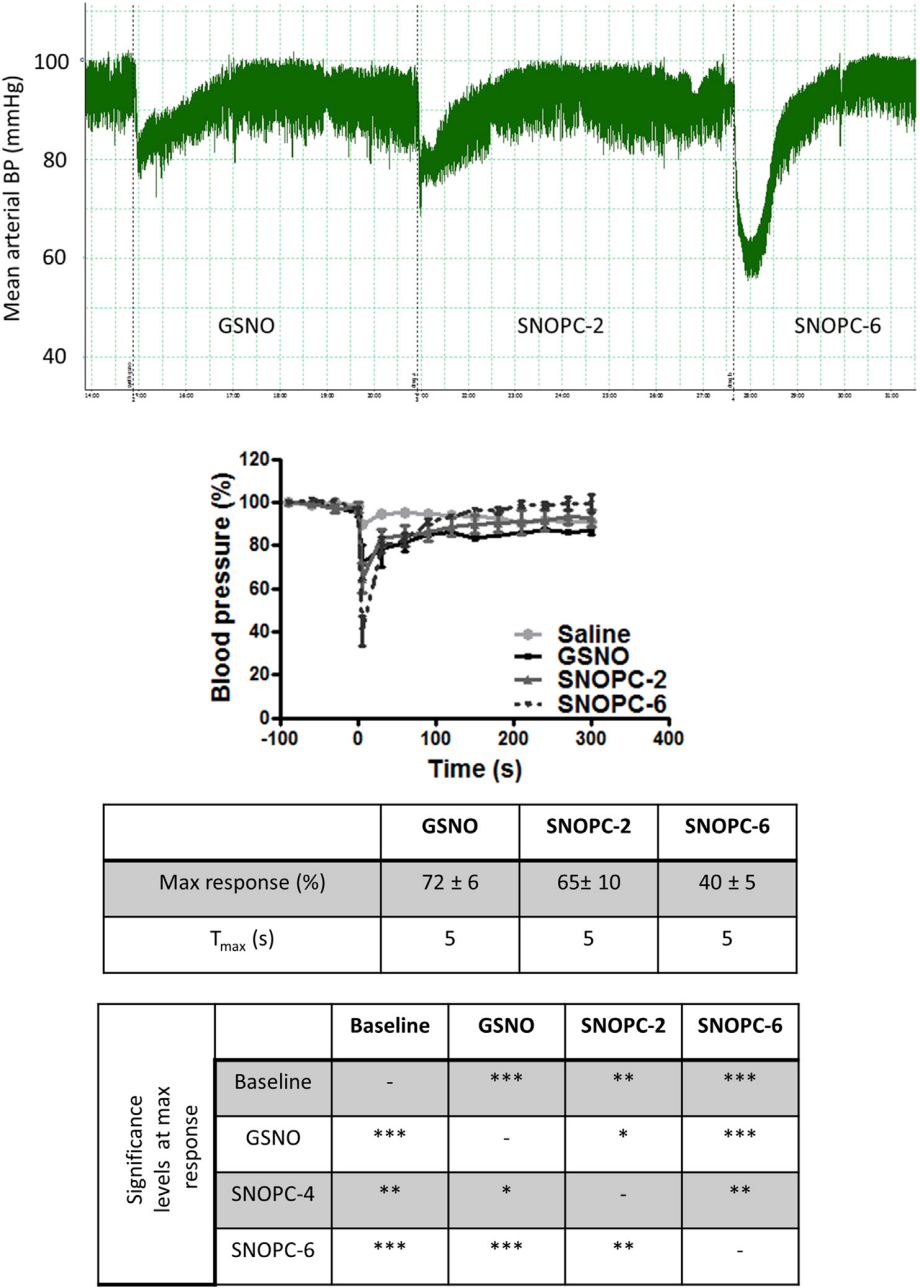
Haemodynamic measurement of hypotensive effects of GSNO, SNOPC-2 and SNOPC-6 after i.v. administration of compounds at equivalent NO doses. (A) A representative trace of mean arterial pressure measured over 5 min post-administration. (B) Mean arterial blood pressure expressed as percentage of pre-administration baseline for all three compounds and the vehicle control. The table includes values of the maximum response achieved (%), time at which the maximum response occurred (t_max_; s) and statistical significance levels. The data shown represent the mean ± SD of n = 3 animals per group (*p < 0.05, **p < 0.01, ***p < 0.001).

Intra venous administration of GSNO and SNOPCs at doses that contained 1 μmol NO/kg body weight resulted in a rapid and transient drop in blood pressure compared to the vehicle control. Fig. 2A depicts a representative example of the haemodynamic traces measured for each compound treatment and Fig. 2B depicts the mean reduction in blood pressure and recovery for each treatment group. What is striking about this data is the significantly greater reduction in blood pressure achieved by treatment with SNOPC-6, despite administration of equivalent doses of NO for all three compounds. This observation contrasts with results from an *in vitro* study of vasodilation in isolated rat aortic rings, where the dose-response curves of SNOPCs and GSNO were nearly identical after treatment with equimolar concentrations of NO [19].

The discrepancy between the *in vitro* and *in vivo* performance of the SNOPCs with regard to vasodilation may be in part explained by the differences in stability of each compound in different environments. *In vitro,* SNOPC decomposition rates (i.e. cleavage of S—NO bonds and ‘release’ of NO from the compound) were found to be compound-dependent with a rank order of: SNOPC-6 > SNOPC-4 >SNOPC-2 > GSNO [19], [20]. *In vitro* RSNO decomposition (in buffer solutions) is primarily driven by photo degradation or transition metal-induced homo- and heterolytic cleavage of the covalent SNO bond [31], [32]. The addition of reducing agents, such as ascorbic acid, may also stimulate decomposition, as well as the presence of other thiols [31], [33]. Transnitrosation, the transfer of NO from a donor RSNO to an acceptor thiol without the release of gaseous NO, also plays a large role in RSNO decomposition and is postulated to be the primary mechanism of RSNO decomposition *in vivo*. This is because other mechanisms, such as photodegradation, rarely occur *in vivo* and transition metals are usually sequestered [31], [32], [34].

Of particular relevance to our *in vivo* observations, is a study by Jourd’heuil et al. [34], who investigated the fate of GSNO and S-nitrosated cysteine (CySNO) in human blood serum and whole blood *ex vivo*. They observed that exogenously administered GSNO and CySNO (10 μmol/L) fully decomposed in both plasma and whole blood within 15 min, whereby ∼85% decomposition took place within in the first 5 min. Half-lives of GSNO and CySNO were ∼120 and 50 s, respectively. Inclusion of transition metal chelators and inhibitors of xanthine oxidase, glutathione peroxidise and γ-glutamyl transpeptidase did not influence the decomposition rate, implying that enzymatic or transition metal-induced pathways of decomposition were not involved. However, amounts of S-nitroso albumin (AlbSNO) increased by approximately 50–70%, providing evidence that (under the conditions tested) transnitrosation from low molecular weight RSNOs to AlbSNO was a primary mechanism of low molecular weight RSNO decomposition in the blood. Another study by Liu et al., also showed that the *in vivo* systemic haemodynamic and *in vitro* vasodilation effects of low molecular weight RSNOs such as GSNO or S-nitroso cysteine occur after equilibration of the NO moiety amongst the plasma thiols (albumin) *via* S-transnitrosylation. This effect was persistent over time where GSNO and S-nitroso cysteine showed a half-life of about 5 min [35].

**Fig. 3:**
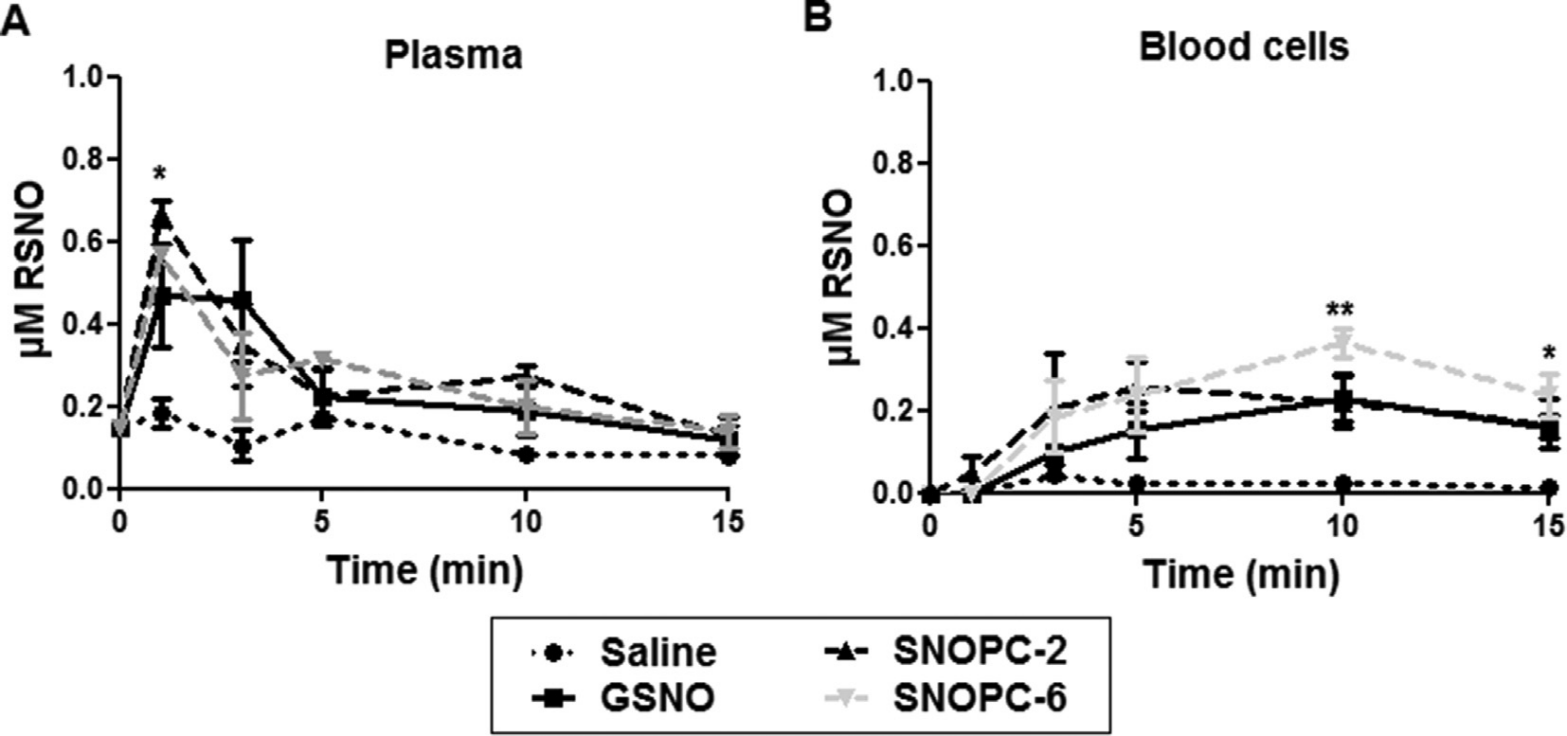
(A) RSNO concentration profile in blood plasma at t = 0–15 min post i.v. administration of GSNO, SNOPC-2, SNOPC-6 (at equivalent molar concentrations of NO) and the vehicle control. (B) RSNO concentration profile in blood cell fraction at t = 0–15 min post i.v. administration of GSNO, SNOPC*-*2, SNOPC-6 (at equivalent molar concentrations of NO) and the vehicle control. The data shown represent the mean ± SD of n = 3 animals per group per time point (*p < 0.05, **p < 0.01).

**Fig. 4:**
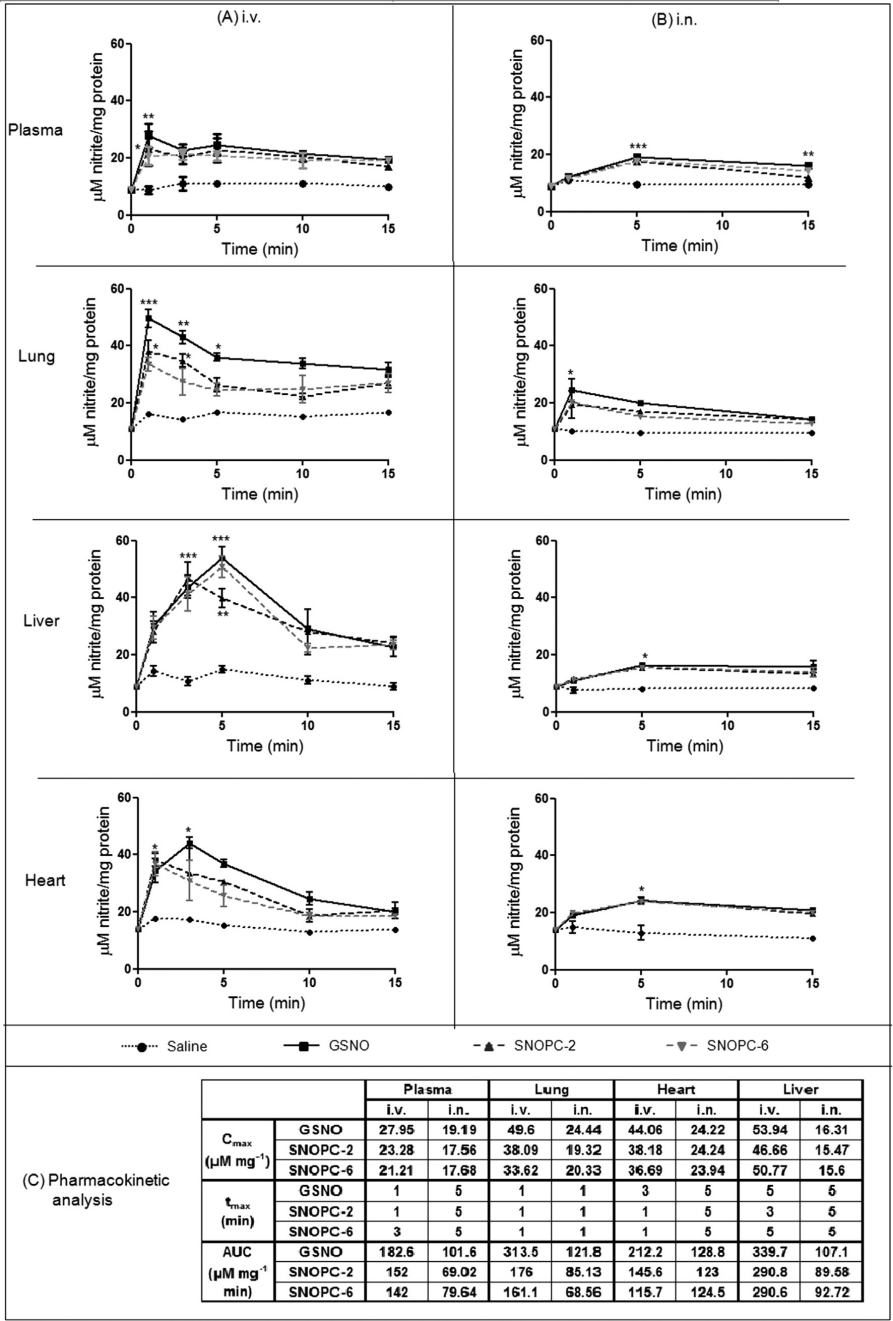
(A) Biodistribution of NOx in selected tissues at t = 0–15 min post i.v. administration of GSNO, SNOPC-2, SNOPC-6 (1 μmol NO/kg) and the vehicle control. (B) Biodistribution of NOx in selected tissues at t = 0–15 min post i.n. administration of GSNO, SNOPC-2, SNOPC-6 (1 μmol NO/kg) and the vehicle control. Data points represent the mean ± SD of n = 3 animals per group per time point (*p < 0.05, **p < 0.01, ***p < 0.001). (C) Pharmacokinetic analysis of NOx in each compartment including comparison of the two administration routes.

Previous *in vitro* studies demonstrated that SNOPCs were more potent transnitrosating agents compared to GSNO [20]. It may be postulated that immediately following i.v. injection, all compounds decomposed *via* transnitrosation reactions with serum albumin to form AlbSNO or other RSNO, providing a circulating storage form of bioactive NO, with SNOPC-6 decomposition occurring most rapidly, followed by SNOPC-2 and GSNO. To test this hypothesis, the RSNO content of fractionated blood (plasma *vs.* blood cells) was investigated. Supporting the observation that RSNO, (including AlbSNO), are an important storage form of NO in blood, a mean baseline value of 0.2 ± 0.05 μmol/L was detected in the plasma compartment (Fig. 3A; in line with literature values for baseline plasma RSNO levels [36], [37]). In comparison, the amount of RSNO in the cellular fraction of blood was nearly undetectable under baseline conditions (Fig. 3B). After treatment with both SNOPCs, a rapid increase in total plasma RSNO was detected, which peaked at t = 1 min and rapidly returned to near baseline levels by t = 5 min (Fig. 3A). Contrary to the stated hypothesis, plasma RSNO levels were not significantly higher after SNOPC-6 treatment compared to those observed following SNOPC-2 treatment. Thus, compound-dependent transnitrosation rates may not alone be sufficient to explain the significant differences in haemodynamic activity between the two SNOPC species. Interestingly, a concurrent elevation in the RSNO levels in the cellular fraction of blood after SNOPC treatment was observed, indicating the dynamic nature of transnitrosation in the *in vivo* environment. The RSNO profiles in plasma and the cellular compartment after GSNO administration provide some evidence of a trend that GSNO may be more stable than either of the SNOPCs and therefore participates in transnitrosation reactions at a slower rate compared to the SNOPCs (Fig. 3A and B); however it should be noted that the NOx curves in plasma were not significantly different from both SNOPC treatment groups (Fig. 4A).

Intravenous bolus injection of AlbSNO (0.001–0.3 μmol/kg) has previously been shown to cause rapid and transient decreases in blood pressure (∼35% reduction compared to baseline) and its activity potentiated by the co-administration of low molecular weight thiols (∼60% reduction) [38], [39], [40]. Thus, a rapid interaction between GSNO/SNOPCs with serum albumin leading to transnitrosation seems a likely intermediary process in the pharmacological activity of these compounds. The exact pathways by which circulating RSNO release or transfer their NO cargo so that the NO or NO metabolite can activate smooth muscle soluble guanylate cyclase (sGC) leading subsequently to vasodilation, remains unknown [34], [41].

### 3.2 I.V. and I.N. administration of GSNO and SNOPCs elevates NOx levels in selected tissues

The quantity and biodistribution of NO metabolites following NO donor administration is often used to evaluate the overall release of NO, as well as assess any targeting effect a delivery system might have [38], [39], [40]. One basic approach is to measure the total levels of NOx in the blood or tissue, which has the advantage of encompassing a wide variety of NO metabolism pathways, most of which ultimately result in formation of nitrites or nitrates. Total levels of NOx were quantified in the heart, liver, lungs, blood plasma and blood cellular fraction after administration of GSNO, SNOPCs, or the vehicle control. Fig. 4A shows total NOx at 1, 3, 5, 10 and 15 min post-i.v. administration in selected organs where NOx levels are significantly increased compared to baseline. Baseline (physiological) levels of NOx were measured to be ∼10 μM nitrite/mg protein for all organs tested (including those not depicted in Fig. 4). I.V. administration of GSNO, SNOPC-2 and SNOPC-6 led to significantly elevated NOx levels in the plasma, lung, heart and liver. At t = 1 min total NOx in the lung was found to be elevated 3.1-, 3.4-, and 4.5-fold higher than the vehicle control group for SNOPC-6, SNOPC-2 and GSNO, respectively. In the other compartments, total NOx concentrations reached a maximum concentration at t = 3 min and returned to near baseline levels by t = 15 min. GSNO-treated animals generally exhibited higher mean AUC values in all compartments compared to other groups (Fig. 4C). It should be noted that the 7-methoxycoumarin fluorescent tag had no significant influence on the NOx biodistribution patterns for all compounds tested (Figure A, supplementary data).

One of the most interesting attributes of SNOPCs as NO delivery systems is the ability to generate a series of compounds with both an increasing number of SNO moieties and controlled increases in molecular weight. This allows a systematic investigation of the impact of oligopeptide size and sequence on the pharmacodynamic and pharmacokinetic profiles of the system using a variety of different administration routes. Because pulmonary delivery of NO donor compounds may be of some interest in diseases such as pulmonary hypertension [21], [22], the biodistribution of NOx was investigated after i.n. administration of GSNO, SNOPC-2 and SNOPC-6 (Fig. 4B). As hypothesized, it was observed that NOx levels and mean AUC values were elevated in the lung tissue compared to i.v. administration over a 15 min period. However, contrary to the original hypothesis, administration of the high molecular weight SNOPC-6 did not result in significant retention of biologically active NO in the lung compared to SNOPC-2 or GSNO. Again, this is likely to be due to the rapid decomposition of SNOPC-6 and transnitrosation reactions occurring in the lung. I.n. delivery of GSNO and the SNOPCs resulted in a mild elevation of NOx in the blood plasma, a moderate increase in liver levels and mild increases in heart levels of NOx indicating that NO metabolites were transported across the respiratory epithelium into the blood or the compounds themselves were able to traverse the respiratory epithelium and enter the blood stream, decomposing to release their NO cargo once there and returning to baseline within 15 min.

Interesting comparisons may be made between the data generated in this study and other similar systems. For example, Katsumi et al. [38], [39] investigated the pharmacodynamics and pharmacokinetics of AlbSNO and pegylated AlbSNO after i.v. administration of 1.5 μmol NO/kg, using GSNO as a control and observed similar results for their GSNO and baseline measurements as seen in this study. They showed that AlbSNO decomposed *in vivo* at a much slower rate than GSNO and SNOPCs as indicated by its higher t_max_ (∼10 min) value for plasma nitrites. Interestingly, pegylation of AlbSNO reduced SNO decomposition *in vivo* achieving a significantly greater AUC for plasma nitrite [30]. It has been demonstrated that ‘caging’ of RSNO in polymer matrices is an effective strategy to stabilize SNO moieties against *in vivo* decomposition. In addition to pegylation of proteins [40], poly ethylene glycol (PEG) hydrogels [42], [43] and interpolymer RSNO complexes composed of poly(vinyl methyl ether co-maleic anhydride)-poly (vinyl pyrrolidone) [8] have been shown to slow RSNO decomposition to achieve NO release over days. Cross linking SNO with alginate was also found to sustain NO release from S-nitrosthiols. Two compounds were synthesized with NO loading 174 ± 21 μmol/g and 468 ± 23 μmol/g respectively. An *ex vivo* permeation study showed that these compounds can exhibit a sustained release for at least 10 h [44] Encapsulation of RSNO in polymeric nanoparticles also provides a promising strategy in sustaining NO release from RSNO. Eudragit^®^ RL was used to prepare GSNO-loaded nanoparticles (GSNO-NP). Their release kinetics showed that C_max_ was reached within 3 h and remained stable till 6 h. In another study, hollow *S*-nitrosothiol polymer nanoparticles as scaffolds for NO release were prepared were the release of NO was controlled by changing the polymer ratio and composition to reach a half-life reaching 225 min [45], [46]. Thus, a viable approach to tuning SNOPC decomposition *in vivo* may be to conjugate the compounds with a suitable polymeric system.

### 3.3 Biodistribution of PC oligopeptides following i.v. administration

**Fig. 5:**
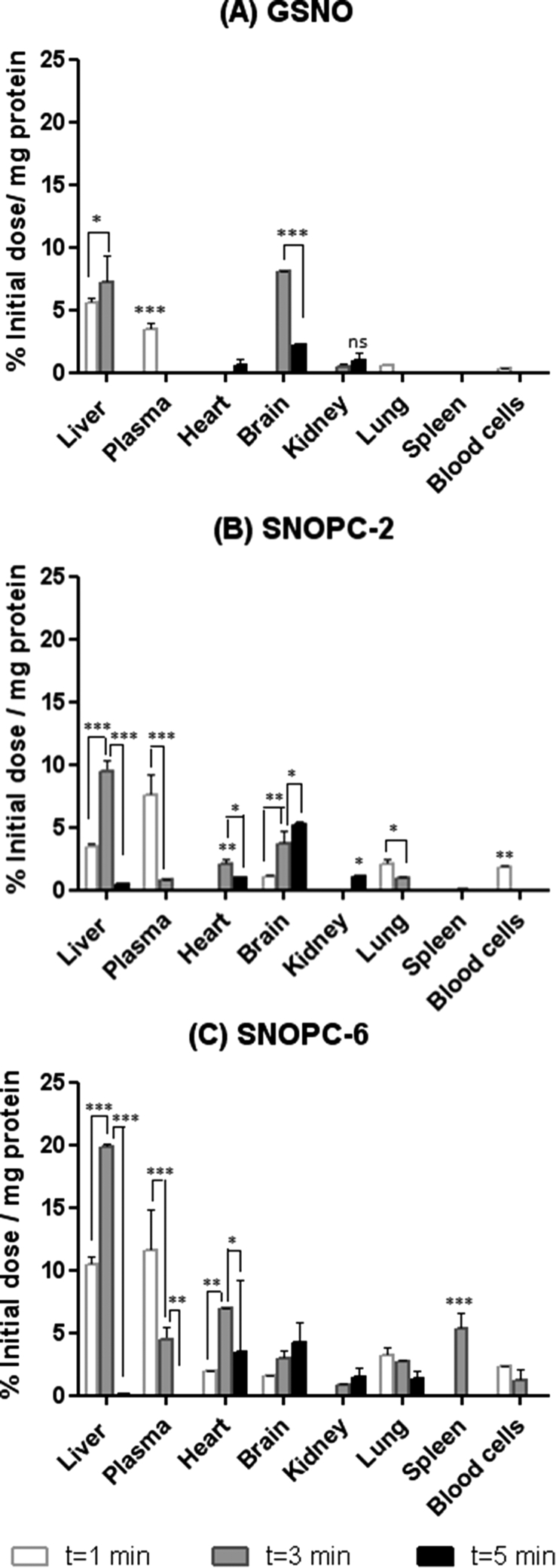
Biodistribution of 7-methoxycoumarin-labelled PCs and GSNO at t = 1–5 min post administration of *GSNO (A), *SNOPC-2 (B), *SNOPC-6 (C). The data shown represent the mean ± SD of n = 3 animals per time point per group (*p < 0.05, **p < 0.01, ***p < 0.001).

In addition to monitoring NOx biodistribution following NO release, it was also of interest to track the fate of the PC oligopeptides after i.v. administration. To evaluate this, GSH, PC-2 and PC-6 were purchased with a 7-methoxycoumarin tag attached to the N-terminus of the peptide chain and nitrosylated according to the same protocol as non-labelled peptides. Fig. 5 shows selected tissues where levels of *GSNO, *SNOPC-2 and *SNOPC-6 associated labels were quantified above the limit of detection at t = 1, 3 and 5 min post-administration of compounds. A molecular weight-dependent biodistribution pattern was observed for these compounds, with overall tissue accumulation increasing as the molecular weight of the peptide increased. The larger oligopeptides showed an increased residence time in the plasma which depleted over time as tissue distribution occurred. Higher concentrations were detected in liver compared with other tissues studied. A further interesting observation was the steady accumulation of SNOPCs in the brain. As the animals were not perfused prior to biodistribution studies, it would be interesting to investigate whether PCs can cross the blood-brain barrier or simply accumulated in the vasculature of the brain. If they are able to cross the blood brain barrier, PCs may be a useful delivery system for other types of compounds in addition to NO, which require blood-brain barrier transport systems.

Little information is available on the susceptibility of PCs to enzymatic degradation, especially by mammalian proteases and peptidases. If analogous to GSH catabolism, PC degradation will likely occur *via* the γ-glutamyl cycle [47]. Thus, exogenously administered PCs may be a substrate of the cell surface enzyme, γ-glutamyl transpeptidase (GGT), which is the initial step in the metabolism of plasma GSH. In humans, GGT is expressed fairly widely, although the highest activity is found in the proximal tubules of the kidneys [47]. GGT activity controls levels of plasma GSH and keeps them at the relatively low concentration of ∼4 μmol/L [48]. A comparison of the biodistribution data for *GSNO and the *SNOPCs provides some limited, indirect evidence that PC distribution and metabolism may differ from GSH. Despite a two to six-fold higher administered dose of GSNO compared to the SNOPCs, *GSNO/*GSH recovery from blood and tissues was generally very low, indicating a rapid and widespread distribution in addition to metabolism by the GGT pathway. Despite the lower administration dose, *SNOPCs/*PCs were recovered at much higher concentrations in blood and tissues, which may indicate that they remain intact for significantly longer periods of time compared to GSH and therefore exhibit a very different tissue accumulation profile. This information is of interest, because very few studies in the literature report biodistribution data of non-targeting oligopeptides (in contrast to peptides such as those containing the integrin-binding RGD sequence [49], [50]). In this respect, PCs may also serve as useful tools for applications beyond NO delivery. For example, it would be interesting to utilise the chelating properties of PCs to bind radiometals for nuclear imaging, which would allow for greater sensitivity and accuracy in biodistribution studies.

## 4 Conclusions

This study investigated the haemodynamic effects of SNOPCs compared with GSNO after i.v. administration and demonstrated that larger SNOPCs such as SNOPC-6 elicit a potent, but transient reduction in blood pressure. The mechanism of this potent effect was hypothesized to result from the excellent transnitrosating properties of SNOPCs. All compounds tested in this study exhibited a corresponding spike of NOx in plasma and selected tissues after both i.v. and i.n. administration although no significant differences in NOx patterns were observed between the compounds. Of particular interest in this study was the observation of selective accumulation of the PC oligopeptides in several organs, notably the brain. Apart from cell targeting peptide sequences, such as RGD, little data exists on the biodistribution patterns of therapeutic oligopeptides with different molecular weights.

Overall, the clinical relevance of this study data lies in a greater understanding of the fundamental SNO chemistry, which will influence RSNO decomposition *in vivo* and lead to compound design with variable NO release properties and thus greater control over pharmacological activity. The study demonstrates that SNOPCs can produce a more potent haemodynamic effect compared to GSNO resulting from changes to the compound structure, i.e. increase in molecular weight and modulation in the stability of SNO groups. It would be feasible that continuous i.v. administration of SNOPCs may be therapeutically valuable in clinical scenarios where a potent vasodilatory effect is required. Additionally, combination of SNOPCs with other drug delivery strategies, such as polymer conjugation, may also allow for modulation of NO release to suit a desired pharmacokinetic profile. Finally, this study also provides interesting general information on non-targeted oligopeptide biodistribution and may be a stimulus to investigate the usefulness of PCs as drug carriers or chelators for radiometals in a wider range of applications.

## Figures and Tables

**Fig. A:**
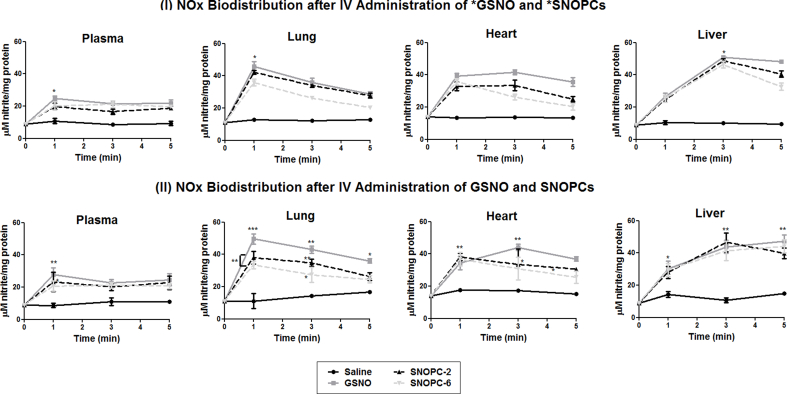
(I) Biodistribution of NOx in selected tissues at t = 0–5 min post i.v. administration of *GSNO, *SNOPC-2, *SNOPC-6 (at equivalent molar concentrations of NO) and the vehicle control. (II) Biodistribution of NOx in selected tissues at t = 0–5 min post i.v. administration of GSNO, SNOPC-2, SNOPC-6 (at equivalent molar concentrations of NO) and the vehicle control. The data shown represent the mean ± SD of n = 3 animals per time point per group (*p < 0.05, **p < 0.01, ***p < 0.001).
